# Common Relationship Between Causality Orientation and the Prefrontal Region in Psychiatric Disorders as Revealed by Diffusional Kurtosis Imaging

**DOI:** 10.7759/cureus.61138

**Published:** 2024-05-26

**Authors:** Miho Ota, Takamasa Noda, Noriko Sato, Kaori Okabe, Kanako Nakazawa, Yoshiko Oshio, Kazuyuki Nakagome

**Affiliations:** 1 Department of Neuropsychiatry, University of Tsukuba, Tsukuba, JPN; 2 Department of Radiology, National Center of Neurology and Psychiatry, Kodaira, JPN; 3 Department of Psychiatry, National Center of Neurology and Psychiatry, Kodaira, JPN; 4 Faculty of Human Sciences, University of Tsukuba, Tsukuba, JPN

**Keywords:** research domain criteria, prefrontal cortex, intrinsic motivation, diffusional kurtosis imaging, causality orientation

## Abstract

Background

Motivation dysregulation is common in several psychiatric disorders. However, little is known about the relationships between motivation and the regional brain areas involved. We evaluated the relationships between brain microstructural features and causality orientation in patients with schizophrenia, major depressive disorder (MDD), and bipolar disorder (BD) using diffusional kurtosis imaging (DKI) techniques.

Methods

Forty patients with MDD, 36 with BD, and 30 with schizophrenia underwent DKI and assessment using the General Causality Orientation Scale (GCOS). We analyzed the DKI index and the GCOS subscales.

Results

The psychiatric patients showed significant positive correlations between the GCOS-autonomy orientation score and the mean kurtosis (MK) values in the prefrontal regions, orbitofrontal regions, and posterior cingulate cortex. When the analyses were performed separately by disease and gender, a positive correlation was found between the GCOS-autonomy orientation score and the MK values in the left prefrontal regions transdiagnostically, especially among female patients with MDD, BD, and schizophrenia.

Conclusions

A similar association between intrinsic motivation and MK value in the left prefrontal cortex was suggested in patients with schizophrenia, MDD, and BD. The commonality of this association among these disorders might lead to the discovery of a new biomarker for psychiatric clinical research.

## Introduction

Self-determination theory focuses on human motivation and personality, highlighting the importance of basic psychological needs as resources for personality development and self-regulation [1]. Causality orientations theory, a component of self-determination theory, describes an individual’s pattern of motivation and behavior. General causality orientations (GCO) are relatively enduring, trait-like characteristics that reflect individuals' beliefs about their ability to promote or cause change and reveal an individual’s motivational pattern [2]. The causality orientations express an internal or external locus of causality, in other words, intrinsic motivation, extrinsic motivation, or amotivation. Intrinsic motivation is driven by internal values, such as interest and enjoyment, whereas extrinsic motivation is generated by external factors, such as reward and punishment. The autonomy orientation is a characteristic of a mature personality and represents a general tendency towards intrinsic motivation [1].

Motivation dysregulation is frequently observed in several disorders, including schizophrenia, bipolar disorder (BD), and major depressive disorder (MDD) [3-5]. Furthermore, previous studies have shown lower autonomy orientation and higher impersonal/amotivated orientations in patients with first-episode psychosis [6], schizophrenia [5], and MDD [7]. As motivation plays a crucial role in modulating treatment effects and is also linked to functional impairment in patients with mental disorders [3,8-9], a greater understanding of the underlying mechanisms of motivation beyond diagnosis is critical to the development of treatment strategies for enhancing functional recovery.

Diffusional kurtosis imaging (DKI) was developed as an extension of diffusion tensor imaging (DTI). The mean kurtosis (MK) value derived from DKI is a dimensionless parameter that reflects changes in the microstructural tissue organization of not only the white matter regions but also the gray matter regions [10]. Some DKI studies have focused on patients with MDD [11], BD [12], and schizophrenia [13]. However, no study has evaluated the relationships between the General Causality Orientation (GCO) in various psychiatric disorders and regional brain microstructures. Previous studies showed that cortico-striatal connections play a central role in deciding goal-directed behaviors based on motivation [14-15]. DKI could provide precise information about the microstructural properties of gray matter [10]. Estimating the relationships between motivation and behavior and regional gray matter microstructure might bring us new findings.

In this study, we analyzed the correlation between the GCOS scores [2] and MK values in patients with MDD, BD, and schizophrenia, respectively, and sought a common GCO-related area among these diseases. A previous study revealed the relationships between intrinsic motivation and activity in the lateral prefrontal region using functional magnetic resonance imaging (fMRI) in patients with schizophrenia [15]. However, no study has evaluated such a relationship in patients with psychiatric diseases beyond diagnosis, despite the clinical need to identify a common measure for the evaluation of motivation in psychiatric diseases. We hypothesized that the GCOS score would be correlated with the MK value in the lateral prefrontal region of patients with psychiatric disorders, based on the previous finding of an association between intrinsic motivation and neuronal activity in this region.

## Materials and methods

Subjects included 40 patients with MDD, 36 with BD, and 30 with schizophrenia. All patients were inpatients at the National Center of Neurology and Psychiatry Hospital, Japan. A consensus diagnosis was made by psychiatrists (TN, KN) according to the criteria in the Diagnostic and Statistical Manual of Mental Disorders, 5th ed. (DSM-5). All patients were rated using the Hamilton Depression Rating Scale-17 (HAM-D17), Young Mania Rating Scale (YMRS), Positive and Negative Syndrome Scale (PANSS), and GCOS. Daily doses of antidepressants were converted to imipramine equivalents, and antipsychotics to chlorpromazine equivalents using published guidelines [16-18]. After the study was explained, written informed consent was obtained from each participant. This study was conducted in accordance with the Declaration of Helsinki and approved by the ethics committee of the National Center of Neurology and Psychiatry, Japan (approval number (A2016-005)).

MRI was performed on a 3-Tesla MR system (Philips Medical Systems, Best, the Netherlands). Three-dimensional (3D) T1-weighted images were used for the morphometric study. 3D T1-weighted images were acquired with the following parameters: repetition time, 7.18 ms; echo time, 3.46 ms; flip angle, 10˚; effective section thickness, 0.6 mm; slab thickness, 180 mm; matrix, 384 × 384; field of view, 261 × 261 mm, yielding 300 contiguous slices through the brain. For diffusional imaging, a multishell protocol was acquired along 16 non-collinear directions at 2 b-values (1000, 2000 s/mm^2^) and one image was acquired without using any diffusion gradient. The acquisition parameters were as follows: echo time, 87 ms; repetition time, 6767 ms; field of view, 240 × 240 mm; matrix = 96 × 96; 60 slices acquired in ascending order. MK maps were calculated and denoised using the Diffusional Kurtosis Estimator (DKE; https://www.nitrc.org/projects/dke). To exclude extraparenchymal noise, we masked the DKI metric maps with the binary mask image made by the segmented gray and white matter images derived from each individual 3D-T1 image. To evaluate MK maps by voxel-based analysis, these images were normalized with the diffeomorphic anatomical registration using the exponentiated Lie (DARTEL) registration method. First, each individual 3D-T1 image was coregistered and resliced to its b = 0 image. Next, the coregistered 3D-T1 image was normalized with DARTEL. Finally, the transformation matrix was applied to the MK maps. Additionally, significant gray matter volume differences among patients with MDD, BD, and schizophrenia [19] necessitate partial volume correction for such diffusional magnetic resonance imaging (dMRI) studies focusing on several diseases. In this study, we used “VoxelStats” for the partial volume correction (https://github.com/sulantha2006/VoxelStats). This program used the segmented individual gray matter volume image for the correction, we normalized each individual 3D-T1 image by VBM8, resliced to 2 x 2 x 2 mm^3^, and smoothed using a 6-mm full-width at half-maximum Gaussian kernel.

GCOS was developed by Deci EL and Ryan RM [2] to evaluate general motivational orientation. This scale identifies three enduring causality orientations: autonomy orientation (the tendency to act according to one's internal interests); controlled orientation (the tendency to be influenced by external events); and impersonal orientation (the tendency to perceive one’s actions as beyond intentional control). Higher scores correspond to stronger tendencies.

ANOVA was performed to determine differences among age groups and the scores of HAMD-17, YMRS, PANSS, and GCOS. The Chi-square test was used to assess gender differences. The significance level of the statistical analysis was set at a p-value < 0.05. Because gender differences in GCOS scores are known [2], we also evaluated gender differences using a two-sample T-test. Statistical analyses were performed using SPSS Statistics for Windows 23.0 software.

For the evaluation of the relationships between GCOS scores and MK, regional gray matter volume, age, and gender of subjects were used as nuisance variables. For multiple comparisons, random field theory (RFT)-based multiple comparison correction was adopted, as recommended by the 'VoxelStats' developer. The cluster threshold was set at p < 0.05 after correction, with initial cluster forming done at p < 0.001 at the voxel level before correction. We first analyzed the correlation between the GCOS scores and the MK value in the group of all participants, and investigated the common relevant areas. Then, we estimated the relationships between the GCOS scores and regional MK values in each diagnostic group. To account for potential gender differences, we estimated the relationships between the GCOS scores and regional MK values by gender, respectively.

## Results

Demographic and clinical characteristics

The demographic and clinical characteristics of the subjects are shown in Table 1. There were no statistically significant differences in gender distribution between the three diagnostic groups. However, the mean age in the MDD group was significantly higher than those in the schizophrenia and bipolar disorder groups. There was also a significant difference in the mean controlled orientation score among the three male groups; the score of the MDD group was significantly lower than that of the schizophrenia group (p < 0.011), but other differences did not reach significant levels. Furthermore, the mean autonomy orientation score of female MDD patients was significantly higher than that of male MDD patients (p = 0.018).

**Table 1:** Characteristics of the study sample. MDD: Major depressive disorder; PANSS: Positive and negative syndrome scale; YMRS: Young Mania Rating Scale; HAMD-17: Hamilton Depression Rating Scale-17: GCOS: General Causality Orientation Scale. ^1 ^Chlorpromazine-equivalent dose ^2 ^Impramine-equivalent dose ^*^There was a significant gender difference. p < 0.05 ^#^There was a significant difference among three groups.

		MDD (mean ± SD)	Bipolar disorder (mean ± SD)	Schizophrenia (mean ± SD)	P-value
Male/Female		20 / 20	18 / 18	12 / 18	0.649
Age (years)		43.2 ± 14.0	37.3 ± 12.7	35.0 ± 9.8	0.019
HAMD-17		17.3 ± 7.3	14.9 ± 8.4	10.0 ± 7.6	0.001^#^
YMRS		0.3 ± 1.4	2.1 ± 3.6	0.8 ± 1.6	< 0.001^#^
PANSS positive		9.0 ± 2.7	9.9 ± 2.9	13.3 ± 4.8	< 0.001^#^
PANSS negative		15.5 ± 5.2	13.1 ± 3.7	16.0 ± 4.8	0.023
PANSS general		32.3 ± 8.7	28.0 ± 5.6	32.8 ± 8.3	0.019
Dose of antipsychotics (mg) ^1^		77.7 ± 173.6	110.8 ± 184.0	449.3 ± 425.1	< 0.001^#^
Dose of antidepressants (mg) ^2^		129.8 ± 113.8	49.0 ± 100.0	25.6 ± 66.0	< 0.001^#^
Dose of lithium (mg)		72.5 ± 184.0	269.4 ± 374.8	0.0 ± 0.0	< 0.001^#^
GCOS					
Autonomy orientation	Male	55.1 ± 13.2*	60.6 ± 8.9	60.0 ± 7.6	0.240
	Female	64.1 ± 9.3*	60.9 ± 9.6	60.5 ± 13.0	0.537
Controlled orientation	Male	37.5 ± 7.6	43.4 ± 9.1	46.3 ± 4.6	0.008^#^
	Female	39.8 ± 10.2	44.8 ± 19.8	42.6 ± 9.6	0.537
Impersonal orientation	Male	49.3 ± 8.7	44.6 ± 8.7	50.8 ± 9.0	0.126
	Female	48.5 ± 9.6	46.2 ± 10.9	49.7 ± 11.0	0.595

Correlation between the MK value and GCOS scores

GCOS Autonomy Orientation (GCOS-A) Value

**Figure 1:**
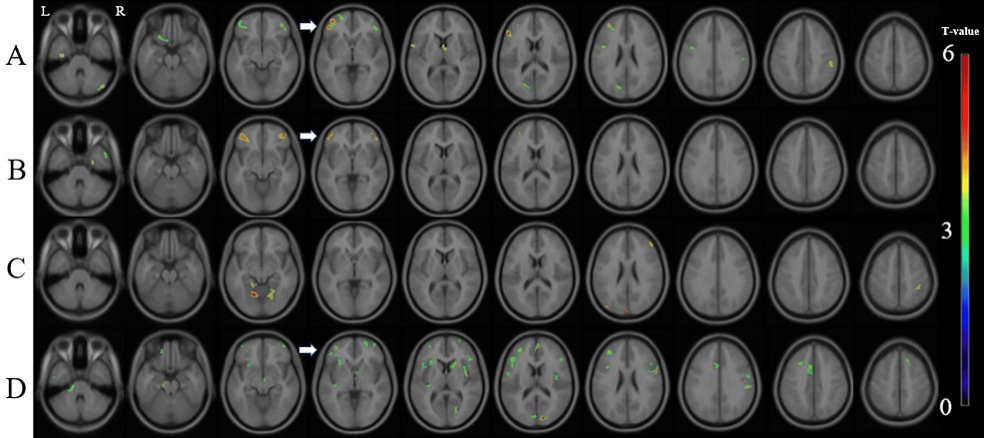
Correlation between the MK value and the GCOS-A score in the male and female patients. There were significant positive correlations between the MK value and the GCOS-A score in the groups of (A) all participants in this study, (B) patients with MDD, (C) patients with BD, and (D) patients with schizophrenia. 'R' stands for right, and 'L' stands for left. The background images are 'avg152T1' images, which are considered anatomically standard images in SPM12. White arrows point to the left prefrontal cortex, which is the common region correlated with the GCOS-A score. GCOS-A: GCOS autonomy orientation; MDD: Major depressive disorder; BD: Bipolar disorder; MK: Mean kurtosis.

We evaluated the correlation between the regional gray matter MK value and GCOS scores in the group of all participants (the patients with MDD, BD, and schizophrenia) by voxel-by-voxel analysis. There was a significant positive correlation between the GCOS-A and the MK value in the bilateral lateral prefrontal cortices, left orbitofrontal cortex, left lingual to posterior cingulate gyrus, and right parietal cortex (Figure 1A). Regarding disease-related differences, there were significant positive correlations between the GCOS-A value and the MK value in the bilateral lateral prefrontal cortices in the patients with MDD (Figure 1B), a significant positive correlation in the bilateral lingual regions and right prefrontal and parietal regions in the BD patients (Figure 1C), and significant positive correlations in the pons, bilateral frontal regions, right caudate, bilateral occipital regions, left anterior cingulate gyrus, and the bilateral insulae in patients with schizophrenia (Figure 1D).

**Figure 2:**
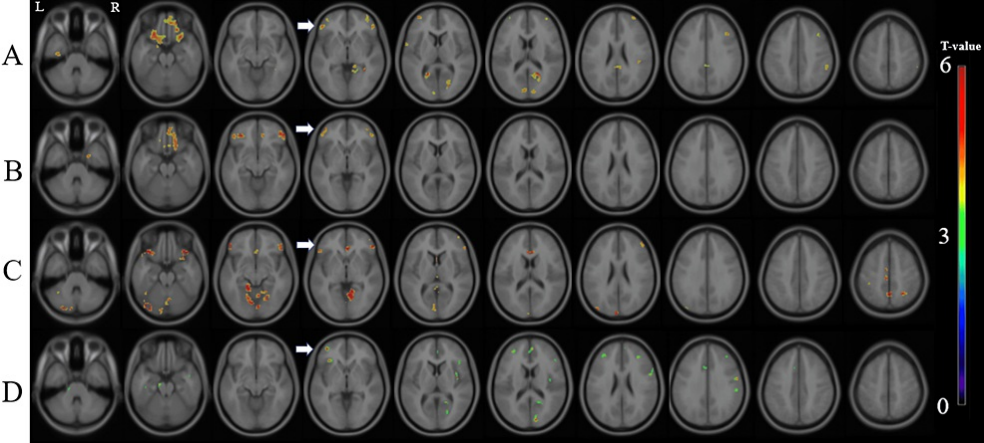
Correlation between the MK value and the GCOS-A score in the female patients. There were significant positive correlations between the MK value and the GCOS-A score in the groups of (A) all female participants in this study, (B) female patients with MDD, (C) female patients with BD, and (D) female patients with schizophrenia. 'R' stands for right, and 'L' stands for left. The background images are 'avg152T1' images, which are considered anatomically standard images in SPM12. White arrows indicate the left prefrontal cortex, which is the common region correlated with the GCOS-A score. GCOS-A: GCOS autonomy orientation; MDD: Major depressive disorder; BD: Bipolar disorder; MK: Mean kurtosis.

To evaluate gender differences, we analyzed the relationships separately in male and female patients. In the group of all female subjects, there was a significant positive correlation between the GCOS-A and the MK value in the left medial temporal cortex, bilateral orbitofrontal regions, bilateral prefrontal cortices, bilateral occipital regions, right parietal region, and posterior cingulate cortex (Figure 2A). In the female patients with MDD, there was a significant positive correlation in the right medial temporal region, bilateral orbitofrontal regions, and bilateral lateral prefrontal cortices (Figure 2B). In the female patients with BD, there was a significant positive correlation in the cerebellum, bilateral lateral prefrontal regions, lingual regions, anterior cingulate cortex, and left occipital region (Figure 2C). In the female patients with schizophrenia, there was a significant positive correlation in the brainstem, bilateral lateral prefrontal regions, right anterior cingulate gyrus, right occipital region, and bilateral insulae (Figure 2D).

**Figure 3:**
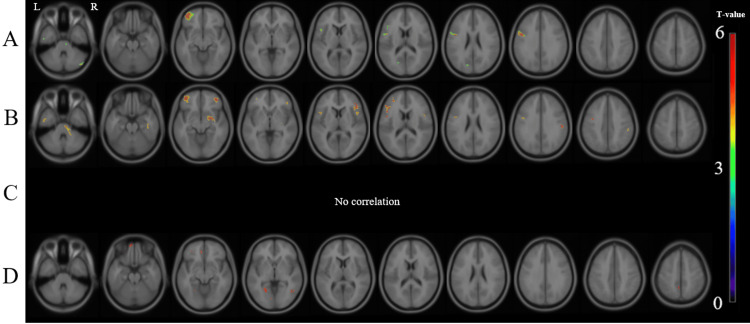
Correlation between the MK value and the GCOS-A score in the male patients. There were significant positive correlations between the MK value and the GCOS-A score in the groups of (A) all male participants in this study, (B) male patients with MDD, (C) male patients with BD, and (D) male patients with schizophrenia. 'R' stands for right, and 'L' stands for left. The background images are 'avg152T1' images, which are considered anatomically standard images in SPM12. GCOS-A: GCOS autonomy orientation; MDD: Major depressive disorder; BD: Bipolar disorder; MK: Mean kurtosis.

In the group of all male subjects, there was a significant positive correlation between the GCOS-A score and the MK value in the left prefrontal region, cerebellum, and left occipital region (Figure 3A). In the male patients with MDD, there was a significant positive correlation in the bilateral lateral prefrontal cortices, brainstem, bilateral temporal regions, right parietal, and right putamen (Figure 3B). In the male patients with BD, there was no significant relationship (Figure 3C). In the male patients with schizophrenia, there was a significant positive correlation in the left orbitofrontal region, left lingual region, left posterior cingulate cortex, and right temporooccipital region (Figure 3D). There was no significant negative correlation between the MK value and GCOS-A scores that was common to all four groups.

In summary, a significant positive correlation between GCOS-A and MK was found in the left prefrontal cortex in both the schizophrenia and MDD groups, as well as in the group of all patients, except for the patients with BD (Figure 1). By gender, the significant positive correlation between GCOS-A and MK in the left prefrontal cortex was reproduced in women with schizophrenia, major depression, and bipolar disorder (Figure 2), while the correlation in men was found only in the group of all patients and the patients with MDD (Figure 3).

GCOS Controlled Orientation Value (GCOS-C)

There was no significant correlation between the GCOS-C score and the MK value in the group of all participants, or in the group of all male participants. Only the group of all female participants showed a significant positive correlation between the GCOS-C score and the MK value in the bilateral thalamus. However, this correlation was not observed among the female patients with MDD, those with BD, and those with schizophrenia (data not shown).

GCOS Impersonal Orientation Value (GCOS-I)

There was a significant positive correlation between the GCOS-I score and the MK value in the left lateral temporal region in the group of all participants. However, this relationship was not observed in the group of all male participants, or in the group of all female participants (data not shown).

## Discussion

We found significant positive correlations between the MK value and the GCOS-A score in the group of all participants. Furthermore, positive correlations between the MK value and GCOS-A score in the left prefrontal region were observed among female patients with MDD, BD, and schizophrenia. To our knowledge, this is the first study to reveal a relationship between the GCOS-A score and brain microstructures using DKI by voxel-by-voxel analysis.

It is known that the prefrontal region and striatum, which compose the frontostriatal loop, are involved in the generation of goal-directed, motivated behavior [14-15]. Previous studies have detected relationships between intrinsic motivation and the activity of the lateral prefrontal cortex in healthy subjects and patients with schizophrenia [15, 20]. Additionally, the striatum, which receives input from midbrain dopaminergic neurons, was responsive to rewards [21] or to internal values even in the absence of an extrinsically rewarding stimulus [22]. Our participants included patients with schizophrenia who were using a variety of antipsychotic medications, and the blockade of dopamine effects induced by these drugs might bring about microstructural changes in the striatum, a dopamine-rich area. A further study enrolling drug-free patients would help to clarify these points.

We observed some positive correlations between the GCOS-A score and MK values in regions from the posterior cingulate cortex to the lingual gyrus, and in the orbitofrontal cortex. A previous study found that the posterior cingulate cortex had anatomical connections to areas involved in reward, memory, and attention [23]. Additionally, a fMRI study detected that activity in the posterior cingulate cortex was well correlated with decision-making based on extrinsic factors [24]. The orbitofrontal cortex also encodes the motivational value of rewards [25], and along with the dorsal striatum, is part of a corticolimbic circuit that encodes incentive value and regulates reward-directed behavior [26]. These findings suggest that the regions we found to be related to the GCOS-A are also subject to extrinsic motivation. One study observed that the GCOS-A score was positively correlated with the GCOS-C score in Japanese populations [27]. It is suggested that motivation comprises several steps on a continuum of relative autonomy. The most autonomous types of motivation are intrinsic in nature, while extrinsic motivation varies from autonomous to controlled, indicating that intrinsic and extrinsic motivations are not clearly separable.

We found a common region that showed a positive correlation between the GCOS-A score and MK values only in the left prefrontal cortex in female patients (Figure 2); however, the same region showed positive correlations between the GCOS-A and MK values in the patient groups of men and women with MDD and schizophrenia, and in all study participants (Figure 1). A previous study detected significant sex-by-diagnosis interactions: male patients with BD had larger left frontal lobes than male controls, whereas the left frontal lobes in female bipolar patients tended to be smaller than in female controls [28]. These points, at least in part, may be related to the gender differences in bipolar patients.

There were some limitations in this study. First, we estimated the relationships between the GCOS score and the regional MK value using 'VoxelStats' to correct for the partial volume effect in the gray matter volume. Thus, we could not check the correlation in the white matter. However, we found relationships between the GCOS score and MK value in the prefrontal cortex not only in patients with schizophrenia, as previously shown [15], but also in those with MDD and BD. This might indicate that our approach was effective for revealing these particular correlations. Second, the sample size was relatively small. A study about intrinsic motivation in schizophrenia showed that the GCOS subscales were well related to the clinical symptoms [5], and further analyses with larger samples to control for clinical symptoms would be useful to confirm our findings. Third, the groups of patients with MDD, BD, or schizophrenia in this study were each medicated with different types of medicine. Since psychotropic drugs affect the survival of neurons, further studies with drug-free participants will be needed to validate our results.

The Research Domain Criteria (RDoC), an initiative by the National Institute of Mental Health (NIMH), is a research framework for investigating psychiatric disorders (https://www.nimh.nih.gov/research/research-funded-by-nimh/rdoc). Traditionally, psychiatric disorders have been conceptualized as disorders that are diagnosed on the basis of a number of symptoms. In that system, because individual psychiatric disorders contain various dysfunctions, the heterogeneity of the individuals included in a single diagnostic entity makes it difficult to elucidate the pathogenesis of psychiatric disorders. The RDoC aims to identify molecular or neural mechanisms of specific domains of mental function rather than disease entities. From these points, the present study investigating a common neural basis underlying GCO patterns in patients with MDD, BD, and schizophrenia follows the framework, and our finding regarding the left prefrontal cortex might shed light on the neural mechanism of one domain, as part of a 'positive valence system'.

## Conclusions

We found significant relationships between the MK value in the left lateral prefrontal region and a tendency toward an autonomy orientation in patients with MDD, BD, and schizophrenia. The fact that we were able to identify an association between clinical symptoms and biomarkers, regardless of diagnosis, is very significant in explaining psychiatric disorders in terms of clinical symptoms, in other words, the aim of RDoC. These results might provide advanced information about the localization of brain function related to GCO transdiagnostically.
